# Three-Dimensional Evaluation on Ecotypic Diversity of Traditional Chinese Medicine: A Case Study of *Artemisia annua* L.

**DOI:** 10.3389/fpls.2017.01225

**Published:** 2017-07-11

**Authors:** Lin Li, Brinckmann A. Josef, Bing Liu, Sihao Zheng, Linfang Huang, Shilin Chen

**Affiliations:** ^1^Institute of Medicinal Plant Development, Chinese Academy of Medical Sciences and Peking Union Medical College Beijing, China; ^2^Sustainability Department, Traditional Medicinals, Sebastopol CA, United States; ^3^Institute of Botany, Chinese Academy of Sciences Beijing, China; ^4^Institute of Chinese Materia Medica, China Academy of Chinese Medical Sciences Beijing, China

**Keywords:** *Artemisia annua*, artemisinin, DNA barcoding, ecotype, ecotypic diversity

## Abstract

Artemisinin is the first-line drug for anti-malaria recommended by the World Health Organization (WHO). As the sole natural plant source of artemisinin, ecotypes of *Artemisia annua* L. vary widely in artemisinin content between nations, and China is the main producing area of *A. annua*. Here we present a three-dimensional evaluation on ecotypic diversity of *A. annua* from 12 main producing areas in China using high-performance liquid chromatography coupled with evaporative light scattering detection (HPLC-ELSD) method, DNA barcoding and ecological analyses. The results indicated that *A. annua* exhibited high ecotypic diversity. *A. annua* grown in the South of the Qinling Mountains-Huaihe River Line had a high artemisinin content, whereas the northern ones were low. Similar pattern was noted in the genetic diversity. The southern *A. annua* had high intraspecific variation in contrast to the northern *A. annua.* In terms of ecological analyses, humidity and sunshine time could be the major limiting ecological factors that affect the accumulation of artemisinin. This is the first reported three-dimensional evaluation integrating chemical, molecular and ecological analyses of the ecotypic diversity of *A. annua*. The work will facilitate exploring the genetic basis of chemical variations and developing strategies for the breeding and cultivation of high quality *A. annua*.

## Introduction

Artemisinin, an endoperoxide sesquiterpene lactone, is effective against drug-resistant malaria. The ‘high efficacy, fast-action and low-toxicity’ artemisinin-based combination therapy (ACT) is recommended by World Health Organization (WHO) as the best choice for acute malaria (Douglas et al., 2010). Researches show that ACT has a cure rate up to 97% against severe falciparum malaria (Dondorp et al., 2005; WHO, 2012). *Artemisia annua* L. (sweet or annual wormwood, member of *Asteraceae* family) is the only plant that synthesizes and accumulates artemisinin (Chinese Pharmacopoeia Commission, 2015; Su and Miller, 2015). *A. annua* is native of Asia, and now it is widely dispersed throughout the world, including America, Australia, Africa, Bulgaria, France, Argentina, Spain and Hungary (Knudsmark Jessing et al., 2014). The plant has been established as crop in agriculture after the statement of WHO, as a valuable component of combinatorial therapy for malaria (Ferreira, 2007). *A. annua* is at present the sole commercial source for artemisinin production since the chemical synthesis and biosynthesis is complicated and uneconomical (Corsello and Garg, 2015). Similar to many other secondary metabolites, trace amounts of artemisinin at a range of 0.01 to 1.4% of dry leaf weight of *A. annua* result in a low ratio of production to demand of this effective drug (Liu et al., 2006). Previous researches show that the Chinese germplasm has higher artemisinin contents than those from Europe, North America, Australia and East Africa (Jain et al., 1996; Zhang et al., 2016).

Chinese scientist Youyou Tu was awarded the 2015 Nobel Prize in Physiology or Medicine for her contribution to artemisinin discovery, which is regarded as a significant breakthrough in 20th century tropical medicine and an important health improvement for people of tropical developing countries (Su and Miller, 2015). Tu screened more than 2,000 Chinese herbal remedies to search for drugs with antimalarial activity. An extract from the wormwood plant *A. annua* proved especially effective while other species of *Artemisia* did not contain artemisinin. In Tu’s artemisinin isolation trials, artemisinin content of samples from Beijing Province, Shandong Province, Liaoning Province, and Anhui Province of China, were low, whereas samples from the Sichuan Province, Hunan Province, Chongqing Province of China had high artemisinin contents (Tu, 2009; Zhang et al., 2016). The result suggested that the artemisinin content was variable in *A. annua* from different geographic origins.

It has been long recognized that plants in general exhibit ecotypic population diversification that reflects influence of environment on a variety of morphological, physiological and chemical traits (Oleksyn et al., 1992; Hancock et al., 2011; Reich et al., 2014). The study of diversification and ultimately speciation is central to evolution and relevant for biology conservation (Weissing et al., 2011). Biogeographic variation has been extensively documented. For instance, Asian cultivated rice consists of two important ecotypes, upland and irrigated, that have, respectively, adapted to either dry land or irrigated cultivation. Upland rice contains abundant drought-related adaptation genes (Lyu et al., 2014). *Moringa oleifera* Lam. ecotypes show a diverse growth performance and leaf mass production (Forster et al., 2015). Our previous research shows that *Panax quinquefolium* L. producing in China can be divided into two chemoecotypes: ginsenoside Rb1-Re from outside Shanhaiguan, and ginsenoside Rg2-Rd from inside Shanhaiguan (Huang et al., 2013). However, although evidence shows that ecotypic variation occurs is abundant (Adams et al., 2016; Perreault-Payette et al., 2017), comprehensive characterization of ecologically important intraspecific variation across biogeographic gradients is lacking, greatly hindering progress in the breeding and cultivation of quality varieties. As the demand for artemisinin remains high worldwide, exploring the chemical and genetic intraspecific variation especially knowledge about its genetic mechanisms for high-yielding become more and more important. Public interest has grown in recent decades; however, the market is not always regulated. Consequently, it is also important to develop accurate and efficient authentication methods of differentiating *A. annua*.

In the present paper, we aim to (i) estimate the chemical variation pattern of *A. annua* from different regions in China using high-performance liquid chromatography coupled with evaporative light scattering detection (HPLC-ELSD), (ii) reveal intraspecific variation of *A. annua* based on DNA barcoding analysis using four regions (ITS2, *psbA-trnH*, *matK* and *rbcL*), and differentiate *A. annua* from its adulterants, and (iii) find possible ecological factors that affect the artemisinin yield. The results will facilitate exploring the genetic basis of chemical variations and developing strategies for the utilization and conservation of *A. annua*.

## Materials and Methods

### Materials

A total of 102 *A. annua* samples for chemical detection were collected from the following 12 main producing areas in China: (1) North of Qinling Mountains-Huaihe River Line: Zuojia city, Jilin province; Dongfeng city, Jilin province; Beijing city; Linyi city, Shandong province; Shangluo city, Shaanxi province; Hanzhong city, Shaanxi province; (2) South of Qinling Mountains-Huaihe River Line: Sangzhi city, Hunan province; Jishou city, Hunan province; Shaodong city, Hunan province; Loudi city, Hunan province; Yunyang city, Chongqing province and Guangzhou city, Guangdong province. The 116 *A. annua* samples for genetic variation analysis were collected from Nanyang city, Henan province, Youyang city, Chongqing province and all the above sampling sites (Nanyang and Youyang are all located in the South of Qinling Mountains-Huaihe River Line). We also downloaded 3 *rbcL* and 3 *matK* sequences of *A. annua* from GenBank. The 80 adulterant samples for the identification analysis included 18 species belonging to 8 genera (*Artemisia, Dendranthema, Asparagus, Quamoclit, Ambrosia, Chrysanthemum, Leonurus, Salsola*). The samples were all cultivars collected in 2015 by Prof. Linfang Huang. The details of these samples are listed in **Table 1**. All the samples were authenticated by Prof. Bing Liu at the Institute of Botany, the Chinese Academy of Sciences (IB-CAS). All corresponding voucher specimens were deposited to the Herbarium of the Institute of Medicinal Plant Development (IMPLAD) at the Chinese Academy of Medical Sciences in Beijing, China.

**Table 1:** Sample information of *Artemisia annua* and its adulterants in this study.

Voucher number	Species	Sampling location	GenBank accession number			
			
			ITS	*psbA-trnH*	*rbcL*	*matK*
ZJ1-ZJ10	*Artemisia annua*	Zuojia city, Jilin province	KX421677-KX421686	KX454535-KX454536 (ZJ1-ZJ2)	∖	∖
DF1-DF9		Dongfeng city, Jilin province	KX421605-KX421613	KX454519-KX454520 (DF1-DF2)	∖	∖
BJ1-BJ11		Beijing city	KX421584-KX421594	KX454515-KX454516 (BJ1-BJ2)	∖	∖
LY1-LY10		Linyi city, Shandong province	KX421649-KX421658	KX454531-KX454532 (LY1-LY2)	∖	∖
SX1-SX5		Shangluo city, Shanxi province	KX421660-KX421664	∖	∖	∖
SXH1-SXH4		Hanzhong city, Shanxi province	KX421665-KX421668	∖	∖	∖
HN1-HN9		Nanyang city, Henan province	KX421623-KX421631	KX454525-KX454526 (HN1-HN2)	∖	∖
YY1-YY13		Yunyang city, Chongqing province	KX421767-KX421779	KX454517-KX454518 (YY1-YY2)	∖	∖
CQYY1-CQYY10		Youyang city, Chongqing province	KX421595-KX421604	KX454533-KX454534 (CQYY1-CQYY2)	∖	∖
SZ1-SZ8		Sangzhi city, Hunan province	KX421669-KX421676	∖	∖	∖
JS1-JS10		Jishou city, Hunan province	KX421632-KX421641	KX454527-KX454528 (JS1-JS2)	∖	∖
LD1-LD7		Loudi city, Hunan province	KX421642-KX421648	KX454529-KX454530 (LD1-LD2)	∖	∖
SD1-SD2		Shaodong city, Hunan province	KX421659 (SD1)	KX454523-KX454524	∖	∖
GZ1-GZ9		Guangzhou city, Guangdong province	KX421614-KX421622	KX454521-KX454522 (GZ1-GZ2)	∖	∖
PS0633MT04		Beijing city	∖	∖	JQ173392	JQ173387
PS0633MT05		Nanning city, Guangxi province	∖	∖	JQ173393	HM989753
PS0633MT08		Chongqing province	∖	∖	JQ173394	HM989754
4W1-4W4		Huludao city, Liaoning province	KX421687-KX421690	∖	∖	∖
6W21-6W22	*Artemisia pubescens*	Chengde city, Hebei province	KX421691-KX421692	∖	∖	∖
7W11-7W12	*Artemisia sieversiana*	Chengde city, Hebei province	KX421693-KX421694	∖	∖	∖
AY1-AY5	*Artemisia argyi*	Shangzhi city, Hunan province	KX421695-KX421699	∖	∖	∖
BB1-BB4	*Artemisia lactiflora*	Enshi city, Hubei province	KX421700-KX421703	∖	∖	∖
BL1-BL2	*Artemisia gmelinii*	Beijing city	KX421704-KX421705	∖	∖	∖
DZ1-DZ5	*Artemisia sieversiana*	Beijing city	KX421706-KX421710	∖	∖	∖
HJ1-HJ7	*Dendranthema chanetii*	Beijing city	KX421711-KX421717	∖	∖	∖
LX1-LX4	*Asparagus schoberioides*	Beijing city	KX421718-KX421721	∖	∖	∖
ML1-ML2	*Artemisia vestita*	Beijing city	KX421722-KX421723	∖	∖	∖
NL1-NL4	*Ipomoea quamoclit*	Beijing city	KX421724-KX421727	∖	∖	∖
TC1-TC3	*Ambrosia artemisiifolia*	Xingcheng city, Liaoning province	KX421728-KX421730	∖	∖	∖
TH1-TH5	*Chrysanthemum coronarium*	Chizhou city, Anhui province	KX421731-KX421735	∖	∖	∖
YA1-YA3, YA21-YA24	*Artemisia lavandulifolia*	Beijing city	KX421736-KX421742	∖	∖	∖
YC1-YC13	*Artemisia capillaris*	Xingcheng city, Liaoning province	KX421743-KX421755	∖	∖	∖
YM1	*Leonurus artemisia*	Beijing city	KX421756	∖	∖	∖
ZM1-3, ZM21-22	*Artemisia scoparia*	Beijing city	KX421757-KX421761	∖	∖	∖
ZMC1-5	*Salsola collina*	Beijing city	KX421762-KX421766	∖	∖	∖


### Artemisinin Determination

In this study, The artemisinin in *A. annua* was quantified by high-performance liquid chromatography coupled with evaporative light scattering detection (HPLC-ELSD). Acetonitrile and methanol (HPLC grade) for HPLC analysis were bought from Fisher (Fair Lawn, NJ, United States). Deionized water was obtained from Millipore Milli-Q purification system (Millipore, Bedford, MA, United States). HPLC-ELSD analyses were performed with a 1525 HPLC System (Waters, United States) equipped with an autosampler, a binary pump, a vacuum degasser, a thermostated column compartment and a evaporative light scattering detection (Alltech, United States).

The dry plant material of *A. annua* were grounded to fine particles and milled to 60 mesh powder before extraction. About 1 g *A. annua* powder sample was extracted in a sealed flask containing 25 mL methanol under room temperature for a given time. Then the suspensions were sonicated with ultrasonic apparatus in a pre-setting procedure for 20 min under 300 W. After extraction, the power was switched off and then the extracts were allowed to cool to room temperature. The extracts were filtered through 0.45 μm nylon membranes prior to injection into HPLC (Hui et al., 2006).

The C18-RP HPLC column (250 mm × 4.6 mm ID; 5.0 μm pore size) was maintained at room temperature. The mobile phase consisted of (A) water-methanol (1:1, v/v) and (B) acetonitrile. The HPLC conditions in the C18 column included the (A):(B) (1:1 v/v) mixture as mobile phase at a flow rate of 1 mL/min. ELSD conditions were optimized at nebulizer-gas flow rate of 2.0 L/min and drift tube temperature of 70°C under the impactor off-mode, and the gain was set at 2 (Avery et al., 1999; Liu C.Z. et al., 2007; Liu J.-L. et al., 2007; Lapkin et al., 2009).

### DNA Extraction, PCR Amplification, and Sequencing

The material specimens were dried by natural methods, and 20 mg of dried plant material was used for DNA extraction. Samples were rubbed for 2 min at a frequency of 30 r/s in a FastPrep bead mill (Retsch MM400, Germany). Genomic DNA was isolated using the Plant Genomic DNA Kit (Tiangen Biotech Co., China), according to the DNA extraction protocol. We made the following modifications to the protocol: chloroform was diluted with isoamyl alcohol (24:1), and the buffer solution GP2 was diluted with isopropanol (same volume). The powdered sample was mixed with 700 μL of 65°C GP1 and 1 μL of β-mercaptoethanol for 10–20 s before the mixture was incubated for 60 min at 65°C. Then, 700 μL of a chloroform: isoamyl alcohol mixture was added, and the solution was centrifuged for 5 min at 12, 000 rpm (∼13400 × *g*). After removed and transferred into a new tube, the supernatant was added 700 μL isopropanol and mixed for 15–20 min. The mixture was centrifuged in CB3 spin columns for 40 s at 12,000 rpm. The filtrate was discarded and 500 μL GD (adding quantitative anhydrous ethanol before use) was added before centrifugation at 12,000 rpm for 40 s. The filtrate was discarded, and 700 μL PW buffer (quantitative anhydrous ethanol was added before use) was used to wash the membrane before centrifugation for 40 s at 12,000 rpm. This step was repeated with 500 μL PW, followed by a final centrifugation step for 2 min at 12,000 rpm to remove the residual wash buffer. The spin column was dried at room temperature for 3–5 min and then centrifuged for 2 min at 12,000 rpm to obtain the total DNA.

General PCR reaction conditions and universal DNA barcode primers were used for the ITS barcodes, as presented in Supplementary Table S1. PCR amplification was performed on 25 μL reaction mixtures containing approximately 20–50 ng of genomic DNA, 8.5 μL of distilled deionized water, 12.5 μL of 2 × *Taq* PCR Master Mix (Beijing TransGen Biotech Co., China), and 1/1 μL of the forward/reverse (F/R) primers (2.5 μM). The reaction mixtures were amplified in a 9700 GeneAmp PCR system (Applied Biosystems, United States). The amplified products were purified using electrophoresis on 1% agarose gels. The purified PCR products were sequenced in both directions by an ABI 3730XL sequencer (Applied Biosystems, United States).

### Climatic Data Collection and Data Analysis

The climatic data were collected from the China meteorological data sharing service system (Website)^1^. The data of 21 climatic factors from 1981 to 2010 were included in our analysis. These climatic factors are listed below: (1) average temperature in January; (2) average temperature in February; (3) average temperature in March; (4) average temperature in April; (5) average temperature in May; (6) average temperature in June; (7) average temperature in July; (8) average temperature in August; (9) average temperature in September; (10) average air pressure; (11) average annual temperature; (12) relative humidity; (13) total annual precipitation; (14) wind speed; (15) average surface temperature; (16) average sunshine time; (17) average vapor pressure; (18) average date of frost; (19) evaporation capacity; (20) longitude; (21) latitude.

The one-way analysis of variance (ANOVA) for the artemisinin content was conducted by SPSS for Windows (release version 22.0; SPSS Institute, Cary, NC, United States). PCA for climatic factors and correlation analysis between artemisinin content and climatic factors were also performed with the SPSS. The level of statistical significance was set at *p*-value < 0.05. The DNA sequencing peak diagrams were obtained and proofread before the contigs were assembled with CodonCode Aligner 5.0.1 (CodonCode Co., United States). All of the sequences were aligned using ClustalW. The NJ trees were constructed based on the Kimura 2-parameter model (Kimura, 1980), and bootstrap tests were conducted with 1,000 repeats to assess the confidence of the phylogenetic relationships by MEGA 6.0 software (Tamura et al., 2013).

## Results

### Chemical Analysis

**FIGURE 1:**
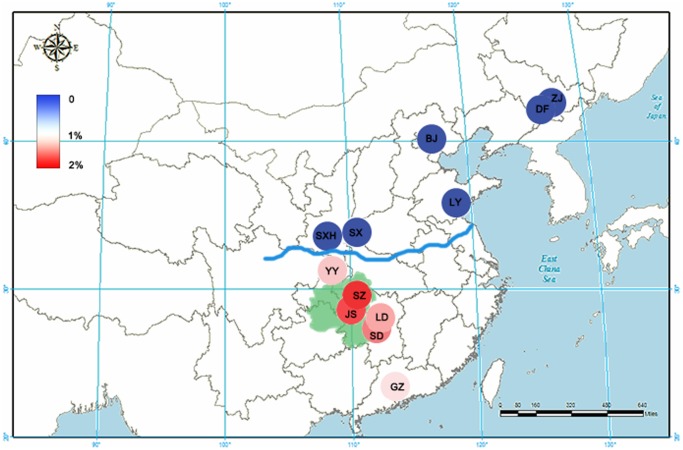
The artemisinin content for *Artemisia annua* from 12 main producing areas in China. The artemisinin contents are depicted with an increasingly stronger blue or red color. The blue line represents the Qinling Mountains-Huaihe River Line. The green zone represents the Wuling Mountain region. ZJ, Zuojia city; DF, Dongfeng city; BJ, Beijing city; LY, Linyi city; SX, Shangluo city; SXH, Hanzhong city; SZ, Sangzhi city; JS, Jishou city; SD, Shaodong city; LD, Loudi city; YY, Yunyang city; GZ, Guangzhou city.

The contents of artemisinin in *A. annua* obtained from different locations were determined and analyzed. As shown in **Figure 1**, *A. annua* grown in the South of the Qinling Mountains-Huaihe River Line (SA) contained an average artemisinin content of 1.52%. In contrast, *A. annua* grown in the North of the Qinling Mountains-Huaihe River Line (NA) had an extremely low artemisinin yield which was below the detection limit. As the center of Wuling Mountain region, samples from Sangzhi and Jishou showed greater artemisinin contents of 1.89 and 1.85%, respectively, forming a distribution pattern that the artemisinin reduced from the Wuling Mountain region to the periphery. A one-way analysis of variance (ANOVA) was employed to perform statistical analysis of the result. It indicated that Group SA and Group NA were significantly different in their content of artemisinin (*p* < 0.05). The results were presented in Supplementary Table S2.

### Genetic Variation Analysis of *A. annua*

To reveal the intraspecific polymorphisms in *A. annua* from different producing areas, the ITS2 and *psbA-trnH* sequences were successfully amplified and sequenced. In addition, another two regions, *rbcL* and *matK*, were downloaded from GenBank. The characteristics of the four barcodes were listed in **Table 2**.

**Table 2:** Sequence characteristics of four selected barcodes.

	ITS	*psbA-trnH*	*matK*	*rbcL*
Number of sequence	116	22	3	3
Amplification efficiency/%	95.87	100.00	∖	∖
Sequencing efficiency/%	100.00	100.00	∖	∖
Length/bp	710–759	457–480	∖	∖
GC content/%	52.53–53.60	28.60–29.47	∖	∖
Number of variable site	18	0	0	0
Number of parsimony informative site	7	0	0	0


#### ITS

**FIGURE 2:**
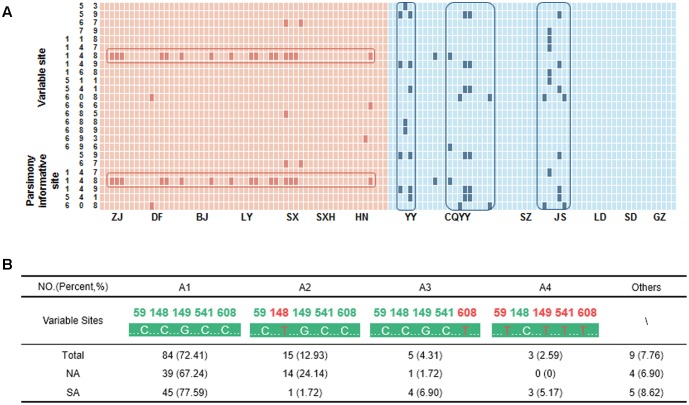
The variable sites and haplotypes for *A. annua*. **(A)** The pink and blue block represent nucleotides in *A. annua* from the South (SA) and North (NA) of the Qinling Mountains-Huaihe River Line, respectively. The deep pink and deep blue block represent the variable sites. HN, Nanyang city; CQYY, Youyang city. **(B)** The main haplotypes of *A. annua*. The pink nucleotides represent the variable sites.

A total of 116 high-quality bidirectional ITS sequences of *A. annua* were aligned. There were 18 variable sites and 7 parsimony-informative sites in ITS region that segregated as 13 sequence haplotypes. The variations were generally nucleotide C to nucleotide T, though few variations were A nucleotides to nucleotide G (**Figure 2A**). The main variants with occurrences greater than 2 were listed in **Figure 2B**. For SA, samples from Wuling Mountain region (Yunyang city, Jishou city, and Youyang city) had high intraspecific variation, which was consistent with the distribution pattern of artemisinin. A1 and A3 represented approximately 85% of the haplotypes, indicating them may be the major ITS haplotype of SA. For NA, a low intraspecific divergence was observed. However, a single nucleotide polymorphism (SNP) sites was found in NA, which was a T nucleotide variation at position 148. The haplotype A2, which represents this variation, coupled with A1 were the dominant haplotype of NA.

#### *psbA-trnH, matK*, and *rbcL*

Regions of the *psbA-trnH* spacer were successfully amplified and sequenced from 22 *A. annua* samples (100%). The results showed that the *psbA-trnH* regions of all the individuals within the *A. annua* were completely conserved. No variable sites were found among them. The same result was observed upon examination of *matK* and *rbcL* sequences of *A. annua* obtained from GenBank, which indicated that the *matK* and *rbcL* genes had a low intraspecific divergence in this species as well.

### Identification Analysis of *A. annua* and Its Adulterants

**FIGURE 3:**
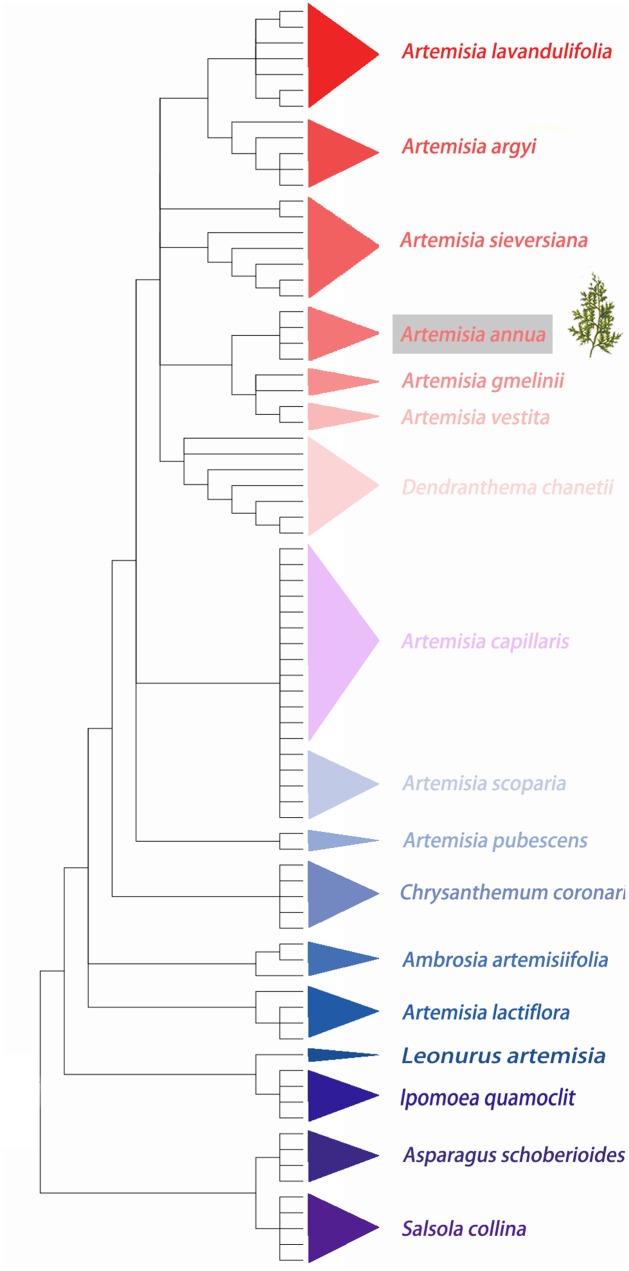
The neighbor-joining (NJ) tree of *A. annua* and its adulterants based on ITS sequence.

A total 80 ITS sequences from 18 species that belonged to 8 genera (*Artemisia, Dendranthema, Asparagus, Quamoclit, Ambrosia, Chrysanthemum, Leonurus*, and *Salsola*) were analyzed. The ITS sequences varied in length from 509 to 731 bp, and the GC content varied from 51.4 to 66.6%. Nearest genetic distance, BLAST and neighbor-joining (NJ) tree methods were used to estimate the capability of species authentication by ITS. Sequence divergences represented by six parameters (**Table 3**) showed that the minimum inter-specific distance was much more than the coalescent depth (maximum intra-specific distance) (**Table 3**), indicating that *A. annua* can be easily distinguished from these 17 adulterants based on the ITS sequences by K2P distance method. NJ tree result also showed that every species clustered into their own clade and *A. annua* can be discriminated from other included taxa (**Figure 3**). Moreover, the successful identification of differences at the species level was 86.3% for BLAST1 and 43.8% for the nearest distance method.

**Table 3:** Analyses of intra- and inter-specific divergence and identification efficiency of ITS sequences.

Parameter		K2P value (± *SD*)
Theta		0.0030 ± 0.0056
Coalescent depth		0.0061 ± 0.0123
All intraspecific distance		0.0037 ± 0.0094
Theta prime		0.0880 ± 0.0619
Minimum interspecific distance		0.0530 ± 0.0740
sAll interspecific distance		0.0876 ± 0.0765
Identification efficiency/%	BLAST1	86.3
	Nearest distance	43.8


### Ecological Factor Analysis

**FIGURE 4:**
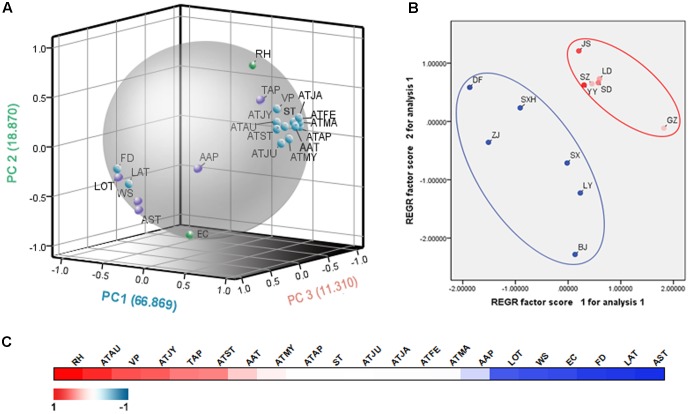
The ecological factor analysis of the 12 main producing areas of *A. annua.* **(A)** The PCA loading plot of the ecological factors. ATJA, average temperature in January; ATFE, average temperature in February; ATMA, average temperature in March; ATAP, average temperature in April; ATMY, average temperature in May; ATJU, average temperature in June; ATJY, average temperature in July; ATAU, average temperature in August; ATST, average temperature in September; AAP, average air pressure; AAT, average annual temperature; RH, relative humidity; TAP, total annual precipitation; WS, wind speed; ST, average surface temperature; AST, average sunshine time; VP, average vapor pressure; FD, average date of frost; EC, evaporation capacity; LOT, longitude; LAT, latitude. **(B)** The PCA score plot of the sampling sites. **(C)** The correlation analysis of ecological factors and artemisinin contents. The correlation coefficients are depicted with an increasingly stronger blue or red color.

To explore the differences in ecology among the sampling sites, a principal component analysis (PCA) was employed. **Figure 4A** showed that the three-component PCA model cumulatively accounted for 97.049% of the variation (PC1, 66.869%; PC2, 18.870%; PC3, 11.310). PC1 was mainly characterized by the average temperature in May, average temperature in June, average temperature in September, average temperature in April. PC2 was correlated with evaporation capacity and relative humidity. Thus, PC1 and PC2 can be defined as temperature factor and humidity factor, respectively. In the score plot (**Figure 4B**), twelve sampling areas were divided into two groups according to their ecological factors. The SA group fell into a large cluster to the top right, whereas the NA group appeared more scattered at the left bottom. The SA group had a higher temperature and humidity than the NA group. The result matched the natural conditions. According to the correlation analysis results (**Figure 4C**), relative humidity demonstrated a significantly positive correlation with artemisinin content, whereas the average sunshine time had a crucially negative influence on the artemisinin content. Furthermore, average temperature in August, average temperature in July, average vapor pressure and latitude were also important ecological factors. Generally, humidity and sunshine time were crucial factors that influenced the artemisinin content.

## Discussion

As the sole plant producing artemisinin, *A. annua* has gained much attention during the last several decades. High-quality raw materials face an increase in demand. Information regarding *A. annua* genetic mechanisms for high-yielding and ecological factors benefiting for producing the plant are central to introduction and cultivation. Given that the variation of secondary metabolite concentration in different plant populations may be largely caused by genetic variation and an interplay of environmental conditions, studies on correlation between chemical constituents variation and genetic diversity can provide better understanding in the ecotypic diversity of medicinal plant (Djabou et al., 2011; Ali et al., 2013; Snyder and Worobo, 2014; Zheng et al., 2014; Xiang et al., 2016a,b). In this paper, a chemical and molecular analysis was combined to reveal the ecotypic population diversification of *A. annua.*

By analyzing the artemisinin content of 102 *A. annua* samples from 12 main producing areas in China, we demonstrated that artemisinin yield varied in different areas of China. A. annua grown in the South of the Qinling Mountains-Huaihe River Line had a high artemisinin content, whereas the northern ones had an extremely low artemisinin yields. It can be generally concluded that the artemisinin content reduced from the Wuling Mountain region to the periphery, which was consistent with our previous studies (Huang et al., 2010). In addition, according to the previous research, the artemisinin yield of some provinces located South of the Qinling Mountains-Huaihe River Line, such as Jiangsu province and Zhejiang province were between 0 and 1.00% (Wei et al., 2008; Xiang et al., 2012). And the artemisinin yield of some provinces located North of the Qinling Mountains-Huaihe River Line, such as Shanxi province and Henan province were as low as 0.10% (Liu J.-L. et al., 2007). This proves our conclusion to some extent.

In previous studies, different types of molecular markers have been used to ascertain DNA polymorphism of *A. annua*. Shi applied RAPD and ISSR to analyze the *A. annua* germplasm resources, and the results showed that *A. annua* gathered from Hubei Province of China had a high intraspecific divergence (Shi et al., 2008). RAPD analysis also indicated genetic variation amongst *A. annua* in the Indian population (Sangwan et al., 1999). By using SRAP markers, Chen *et al.* found that the *A. annua* germplasm resources in natural populations of China had an extremely abundant genetic diversity, especially the *A. annua* germplasm in Chongqing Province (Chen et al., 2011). In EST-SSR analysis, a variety of motifs was detected (Wang et al., 2012). In recent years, DNA barcoding has been suggested as a useful molecular marker technique to complement traditional taxonomic methods for biodiversity inventory construction and fast species identification (Hollingsworth, 2011; Stoeckle et al., 2011; Little and Jeanson, 2013; Nithaniyal et al., 2014; Wu et al., 2015). In the present study, SA contained a high intraspecific divergence in the ITS regions, especially samples from Wuling Mountain region. The NA had a low intraspecific divergence, which was consistent with the distribution pattern of artemisinin. However, a significantly low sequence divergence was observed in the three chloroplast regions investigated (*psbA-trnH, matK, and rbcL*). One of the possible reasons is that chloroplast genes evolve slowly compared with nuclear genes. The conservation of chloroplast DNA sequences can ensure comparability among groups. Consequently, *rbcL* sequence is generally applied to the classification of higher taxa above the family level, while *matK* and *psbA-trnH* sequences are suitable for identifications at the genus and species level (Hebert and Gregory, 2005; Chase et al., 2007; Kress and Erickson, 2007). The ITS sequence has different copy numbers in different taxa, resulting in high existence of variants in the genome (Alvarez and Wendel, 2003; Nieto Feliner and Rossello, 2007). The abundant variations in ITS can be used in the classification of lower-level taxa (genera, species, subspecies) as far as population level. Our research has confirmed it to a certain extent. Although variations among *A. annua* from different sampling sites are insufficient to divide into species or subspecies, it reflected an internal variability and DNA polymorphism in *A. annua* and provide information for the optimal utilization and conservation of *A. annua* germplasm resource. This work also illustrates the current limitations in the applicability of DNA barcoding to taxonomic surveys. Therefore, further studies are required to protocol improvement and development, particularly design of novel universal primers to extend the barcoding to a broader coverage of plant species.

To identify anti-malaria drugs, *A. annua* was filtrated from more than 2,000 Chinese herbal remedies. The other species from Artemisia have been proved not to contain artemisinin. After a large-scale national survey, Tu found five main commercial adulterants of *A. annua: A. apiacea, A. scoparia, A. capillaries, A. japonica*, and *A. eriopoda* (Dondorp et al., 2005). The adulterants share similar morphological characteristics with *A. annua*, although they show different pharmacological properties and medicinal values (Xiang et al., 2016b). In the present study, the barcoding ITS effectively identified *A. annua* from other species of *Artemisia*, which can significantly reduce the adverse effects on the health of consumers caused by herbal product adulteration and contamination in the traditional herbal medicine market.

The plant content of artemisinin is partly determined by biotic factors, such as germplasm, but also by abiotic factors, such as climate (Hancock et al., 2011). In our PCA analysis, the sampling sites belonging to Wuling Mountain region aggregated closely due to their unique geographical factors. As disclosed in the correlation analysis, humidity and sunshine time were the most distinct positive and negative ecological factors for *A. annua*, respectively. Temperature, especially temperature in August and July also played an important role in the accumulation of artemisinin. Wuling Mountain region lies in a subtropical monsoon climate that provides suitable temperature and abundant precipitation. Because of the mountains, the region is foggy and lacks sunshine. Consequently, the unique environment facilitates the accumulation of artemisinin. As an annual plant, *A. annua* blossoms in September. The production of artemisinin usually peaks with flowering. Thus, the plant is very responsive to the temperature in August and July. The result is consistent with our previous study (Huang et al., 2010). Qinling Mountains-Huaihe River Line is one of the most important geographical boundaries in China. The significance of the geographic dividing line is reflected in the boundary of (1) North China and South China; (2) humid areas and semi humid areas; (3) subtropical zones and warm temperate zones; and (4) paddy fields and dry land, namely, rice and wheat main production boundary. Ancient Chinese wisdom indicated that for tangerine plants with similar leaves, tangerine plants grown in the South of Huaihe River would produce tangerines, whereas tangerine plants grown in the North of Huaihe River would produce bitter oranges. China is vast in territory with diversified landforms and mountain ranges and, hence, varying in weather patterns. This diversity of natural ecosystems, climatic conditions and edaphic traits produce abundant diversities among botanical and germplasm resources and contribute to different quality levels of medicinal plants. Environmental stress is an external factor that affects the formation of certain geo-authentic herbs (Zhao et al., 2012). The favorable ecological factors indicated the ecological requirements of *A. annua* and provided useful guidelines for *in vitro* and *ex situ* cultivation.

## Conclusion

In this study, a three-dimensional method that integrated chemical detection, molecular identification and ecological analyses was developed for evaluating the ecotypic diversity of *A. annua* for the first time. By this approach, high ecotypic diversity of *A. annua* was observed. We demonstrated that the artemisinin content reduced from the Wuling Mountain region to the periphery. We also found high level of intraspecific genetic diversity among *A. annua* grown in the South of the Qinling Mountains-Huaihe River Line. Humidity and sunshine time were the essential ecological factors influencing the artemisinin content. This work documents the climatic adaptation of *A. annua* and provides useful guidelines for *in vitro* and *ex situ* cultivation. Our intensive research on transcriptomics to test the key enzymes in the biosynthesis of artemisinin are ongoing.

## Author Contributions

BL and LH collected and authenticated all the samples. LH, LL, SZ, BJ, and SC contributed to the study design. LL and SZ conducted the experiments. LL analyzed all the data and wrote the manuscript. All authors have read and approved the final manuscript.

## Conflict of Interest Statement

The authors declare that the research was conducted in the absence of any commercial or financial relationships that could be construed as a potential conflict of interest.

## Footnotes

1 http://data.cma.cn/
