# Supplementary material for: Three-Dimensional Evaluation on Ecotypic Diversity of Traditional Chinese Medicine: A Case Study of Artemisia annua L
Source: Front Plant Sci. 2017 Jul 11;8:1225. doi: 10.3389/fpls.2017.01225 (PMC5504922; doi:10.3389/fpls.2017.01225)
Supplement: Supplementary file 1 [file Table_1.DOCX]

Table S1 Primers and PCR reaction conditions

| Primer name | Primer sequences（5’-3’） | PCR reaction condition |
| --- | --- | --- |
| ITS 4R | TCCTCCGCTTATTGATATGC | 94℃ 5min； |
| 5F | GGAAGTAAAAGTCGTAACAAGG | 94℃ 1min，50℃ 1min，  72℃ 1.5min+3s/cycle，30cycles；  72℃ 7min； |
| *psb*A fwd PA | GTTATGCATGAACGTAATGCTC | 94℃ 4min； |
| *trn*H rev TH | CGCGCATGGTGGATTCACAATCC | 94℃ 30s，55℃ 1min，  72℃ 1min，35 cycles；  72℃ 10min； |
