# Supplementary material for: Three-Dimensional Evaluation on Ecotypic Diversity of Traditional Chinese Medicine: A Case Study of Artemisia annua L
Source: Front Plant Sci. 2017 Jul 11;8:1225. doi: 10.3389/fpls.2017.01225 (PMC5504922; doi:10.3389/fpls.2017.01225)
Supplement: Supplementary file 2 [file Table_2.DOCX]

Table S2. ANOVA results for the artemisinin content of SA

|  | [Quadratic](C:/Users/Dell-pc/AppData/Local/Yodao/DeskDict/frame/20160310211433/javascript:void(0);) [sum](C:/Users/Dell-pc/AppData/Local/Yodao/DeskDict/frame/20160310211433/javascript:void(0);) | df | [Mean](C:/Users/Dell-pc/AppData/Local/Yodao/DeskDict/frame/20160310211433/javascript:void(0);) [square](C:/Users/Dell-pc/AppData/Local/Yodao/DeskDict/frame/20160310211433/javascript:void(0);) | F | p-Value |
| --- | --- | --- | --- | --- | --- |
| Inter-group | 4.605 | 5 | 0.921 | 7.876 | <0.05 |
| Intra-group | 5.496 | 47 | 0.117 |  |  |
| Total | 10.101 | 52 |  |  |  |
